# Determination of the creep behavior of potato tubers during storage period by means of uniaxial and triaxial creep tests

**DOI:** 10.1002/fsn3.1468

**Published:** 2020-02-17

**Authors:** Gholamhossein Shahgholi, Meysam Latifi, Behrooz Imani, Niusha Farrokhi

**Affiliations:** ^1^ Department of Biosystems Engineering Faculty of Agriculture and Natural Resources University of Mohaghegh Ardabili Ardabil Iran

**Keywords:** creep, potato, triaxial, uniaxial

## Abstract

The analysis of static forces is essential in the storage of agricultural products because these forces are applied in fruits, vegetables, or grains that can lead to their permanent deformation. This mechanical damage increases the product waste. Given that the potato tuber is surrounded by other tubers in the storage, then every tuber is pressurized from all sides or so‐called triaxial stress. To measure the deformation of the specimens, a device was developed to conduct uniaxial and triaxial creep tests on the viscoelastic agricultural products (e.g., potatoes). Accordingly, the creep experiments were performed on potato samples for 5, 30, 60, 120, 240, 360, 420, and 480 min under the constant load of 72.5 N at 3 and 11 replications via uniaxial and triaxial tests, respectively. The results showed that in the creep test, the loading time was significant on the deformation of the specimens at the 0.05 level of probability. Triaxial test showed that the tuber deformation and strain of top layer were more than the middle and bottom layers. The results showed that the deformation of tubers under uniaxial test was more than triaxial one.

## Graphical abstract

This paper compares potato creep using uniaxial and triaxial methods.

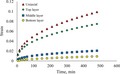

## 1 INTRODUCTION

The damages, caused by the static loads on the stored agricultural products, are of great importance, as they affect the fruits, vegetables, or grains. Some types of fruits and vegetables, such as apples, potatoes, tomatoes, are rapidly damaged in such conditions. To conduct a mechanical analysis, it is important to know how the components interact with each other in addition to the mechanical properties of the product. Forces, transmitted from one fruit to another in a crop, can lead to permanent deformation at the points of contact on the fruit or vegetable, and cause mechanical damage (Masoumi, Tabatabaeefar, & Borghaee, 2005).

The reduction of fruits and vegetables waste will require modifying the production structure of these crops from farm to storage during harvest, transportation, and marketing. It is an effective way to improve the technical knowledge of producers, fruit and vegetable managers, retailers, and ultimately consumers in this direction (Rahemi, 1994).

In Iran, around 3.3 million tons of potatoes are produced annually and 2 million tons are stored in stock. At least 600,000 tons of stored products are wasted due to the unstable storage conditions (Hashemian, 2013). In the stacked storage method, the product is subjected to a constant load due to the weight of the top layers for several months. Therefore, the creep test can be used to simulate this storage. In the creep test, a constant stress is applied to the specimen, the strain is examined as a function of time, and the strain diagram is gradually determined by the viscoelastic properties of the specimen (Alvarez, Saunders, & Vincent, 2000).

Tissue loading was not significantly affected by any of the mechanical parameters. This result can be attributed to the single‐cell structure of the middle parenchymal tissue. It also shows that the cells of the middle tissue of the potato consist of a nearly homogeneous and isotropic structure. This result is confirmed by the morphological examination of the cellular structure in the parenchymal tissue (Mohsenin, 1986).

There was an investigation via the creep test on the changes in the rheological and mechanical properties of pears during the storage for 4 weeks. The results showed that changes in rheology properties can be described as a function of storage time. Also, it was found that to estimate the quality of the stored product, the use of change in rheology properties during storage was better than the measurement of physical properties (Amer Eissa & Alghannam, 2012).

In another study, changes in rheology properties of potatoes during storage were modeled under constant and variable conditions. Also, the parameters of elasticity and viscosity of potato were determined via the creep and axial compression test. The Burger Four‐Element Model could accurately simulate the creep curve. With the increment in the shelf‐life of the modulus of elasticity, the elastic and viscous properties of potato except the Newtonian flow viscosity under both constant and variable conditions were significantly reduced (Solomona & Jindal, 2007). Soliman and EL‐Sayed (2017) studied the creep behavior of Lady Rosetta potato tuber variety of sand and black soil cultivars under different storage treatments. The fresh‐harvested tubers tend to be very brittle, and the creep apparatuses were developed via a digital micrometer, enabled to record the data into the computer and instantaneous reading of deformation at the constant stress of 62.45 kPa during 60 min. The creep curves were analyzed, and the constants related to Burger rheological models were determined. For fresh potato tubers, all rheological model constants were slightly increased with tuber mass and rheological model constants of black soil cultivar were more than that for sand soil cultivar. The retardation of the rheological model was found to be constant around 621 ± 5 s for each of sand and black soil cultivars*.* The properties of food rheology (e.g., modulus of elasticity, deformation index, viscosity, and flow index) were determined and modeled by investigation via creep and relaxation test. The results showed that the proposed mathematical models (e.g., an elastic member [spring], a viscose member [damper], and a plastic member) were able to determine the rheological properties of the studied materials (Myhan, Białobrzewski, & Markowski, 2012). Rady and Soliman (2017) investigated the effect of surface type and angle, drop height, and number of impacts on the creep behavior of Lady Rosetta potato cultivar. The impact recording device was used to obtain the coefficient of restitution and to calculate the absorbed energy for steel sheet, steel rods, rubber‐coated steel rods, and two‐layer potato surfaces. The four‐element Burger model was used to simulate the creep behavior of samples. The results showed that there was a significant effect of drop height, surface angle, and number of impact on the creep parameters. Higher parameters values (i.e., lower incident strain) were associated with steel rods and steel sheet surfaces, dropping tubers from 100 cm, and dropping tubers for 5 and 10 times. Moreover, the two‐layer potato surface was found to cause the lowest strain values to the dropped tubers compared with other tested surfaces.

Given that the tuber is surrounded by other tubers during storage, any glands are compressed from all sides or subjected to a triaxial stress. In most of the previous studies, however, the sample was tested uniaxially and unbound. Accordingly, the triaxial creep test is close to the actual condition of the product in storage compared to the uniaxial test. In uniaxial mode, the stress is applied from the top and bottom, and the sample is free from other sides, while in storage condition, the tuber is surrounded by other tubers. By applying the stress from the sides, the strain is prevented from excessive loading. Therefore, the strain is expected to be less in triaxial loading in all cases than uniaxial one. Also in some studies, the product samples are produced and put in a metal cylinder under loading, enclosed inside a metal cylinder (Daraee & Hemmat, 2013). As the metal cylinder is rigid and its inside is filled with viscoelastic material, then the potato sample merely deforms, while in storage condition, both tubers share the deformation in contact area. To measure the deformation of the specimens, a device was developed that could perform the potato creep uniaxially and triaxially. The aim of study was to measure and compare the creep of potato samples via the uniaxial and triaxial methods. It also investigated the effect of loading time on the potato tuber creep under a constant load of storage conditions.

## 2 MATERIALS AND METHODS

### 2.1 Measurement of physical properties

The tuber samples (*Agria variety*), harvested in September 15, 2018, were taken from Ardabil Agricultural Research Center (38°12′46″N, 48° 17′42.2″E, and 1,350 m above sea level) and were kept in the refrigerator for a week. It was necessary to determine the percentage of moisture as it is one of the factors affecting the deformation of the specimens. To this end, five samples of potato were randomly selected. The crushed specimens were weighed by digital scale with a precision of 0.01 g. Then, they were placed in an oven at 70°C for 72 hr (AOAC, 2005). After 72 hr, the weight of specimens was measured immediately. Equation 1 was used to determine the moisture content of the samples.

(1) $$\text{Mw}=\frac{\text{Ww}}{\text{Ww}+\text{Wd}}\times 100$$

where: Ww and Wd are the water weights wet and dried samples, g.

On average, the moisture of the samples was 80.85% based on fresh weight. To measure the actual specific density, five potatoes were randomly selected. The weight and the volume of the specimens were measured by fluid displacement with water. True density of the samples was calculated by the Equation 2 (Jahanbakhshi, Abbaspour‐Gilandeh, Ghamari, & Heidarbeigi, 2019).

(2) ρ=M/V

where: *ρ*: true density, kg/m^3^; *M*: weight of sample, kg; *V*: sample volume, m^3^.

The calculated density of potato was 1,045 kg/m^3^ on average. About 30 samples of whole tubers were randomly selected whose dimensions were measured with via Vernier caliper with an accuracy of 0.01 mm. Also, the sphericity, arithmetic, and geometric mean diameter and surface area of the samples were calculated (Moradi, Mousavi Khaneghah, Parvaresh, & Balanian, 2019). Table 1 presents some characteristics of the tested potato.

**Table 1:** Average value of some parameters of tested samples

Characteristics	Equation	Value
Major diameter, mm	*a*	71.146
Intermediate diameter, mm	*b*	61.139
Minor diameter, mm	*c*	50.561
Arithmetic mean diameter, mm	$D_{a}=\frac{L+W+T}{3}$	61.091
The geometric mean diameter, mm	$D_{g}=\sqrt[{3}]{L.W.T}$	60.434
Sphericity coefficient	$\varphi =\frac{\sqrt[{3}]{L.W.T}}{L}$	85.193
Surface area, cm^2^	$S=\pi D_{g}^{2}$	114.679
Volume, cm^3^	$V=\frac{\pi .L.W.T}{6}$	115.097

**Figure 1:**
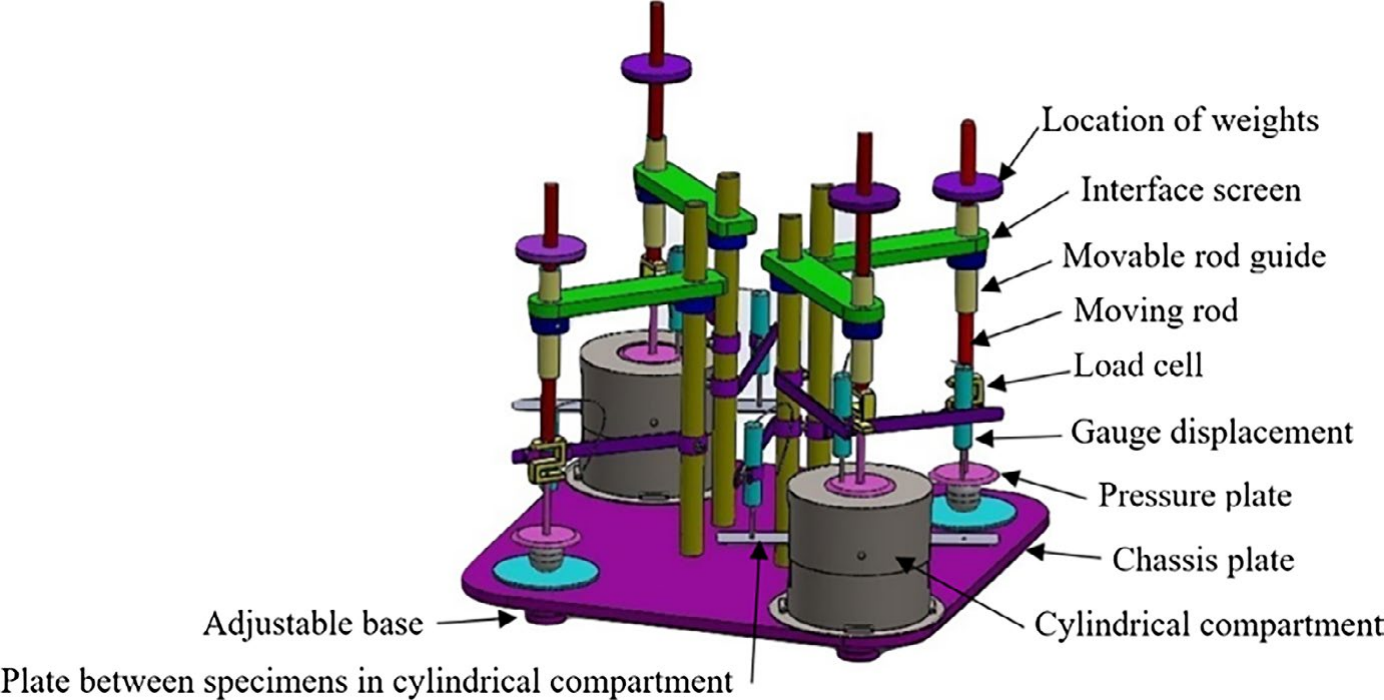
Overview of the developed device

The uniaxial and triaxial creep tests were used to determine the strain rate of potatoes relative to time in storage. The device has four independent units; two of which are designed for triaxial creep test and two for the uniaxial creep test (Figure 1).

**Figure 2:**
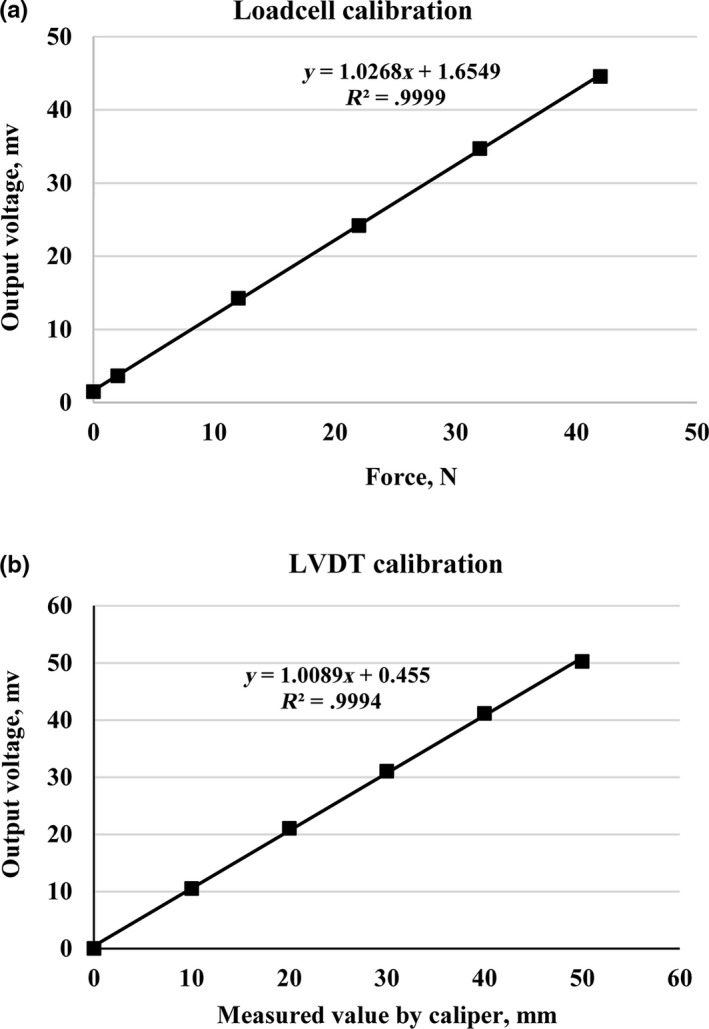
Calibration of the load cell and displacement transducer

The units for the triaxial test comprised a cylinder with 180 mm high and 200 mm diameter which had a capacity of three layers of medium‐sized potatoes or tomatoes. Three displacement transducers (Linear potentiometer or LVDT) with the capacity 50 mm and a precision of 0.001 mm were used to measure the displacement of the product under loading. Also, 100 kg of load cell was used to measure the axial force applied to the products. The data logger, made by Data Taker Company of Australia, and laptop were used to record the data. Before using any measuring device, load cells were calibrated by the specified weights and the displacement transducers by a caliper with an accuracy of 0.01 mm (Figure 2).

**Figure 3:**
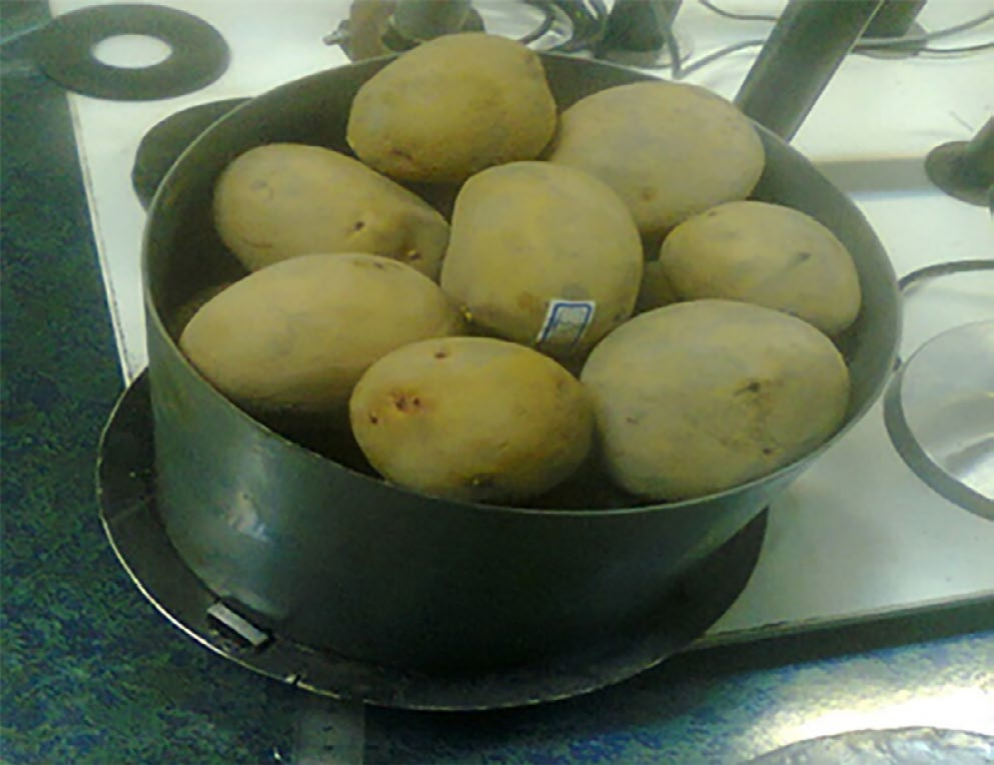
Samples of potatoes inside the cylinder

**Figure 4:**
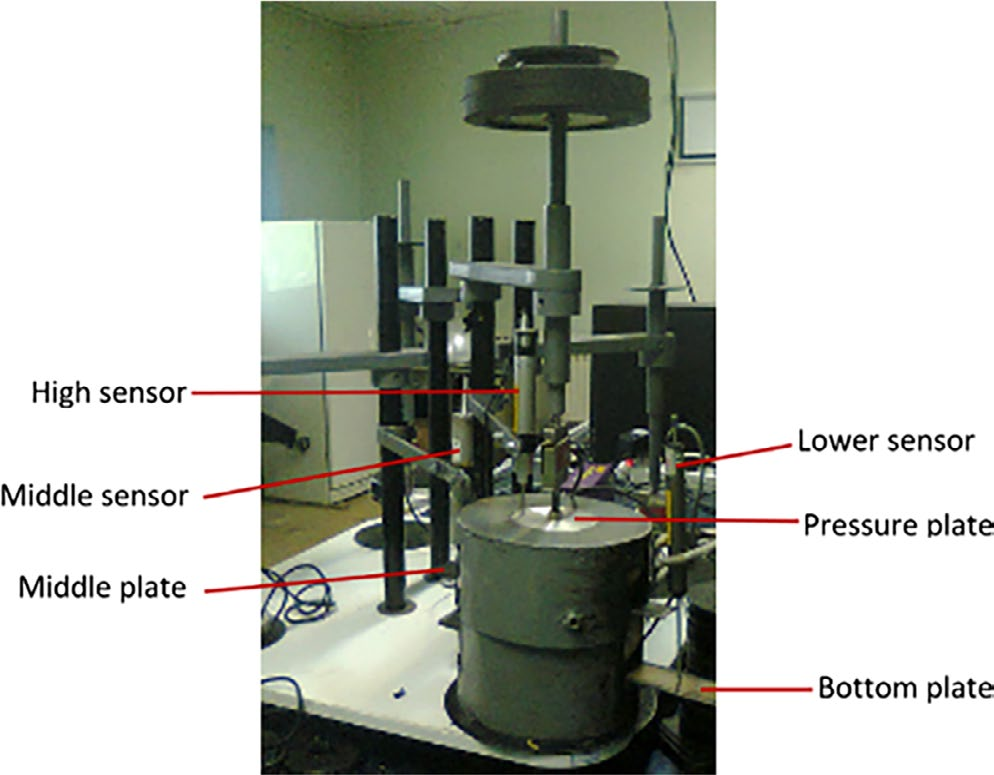
How to place the displacement sensors on the plates in the triaxial test

Twenty medium‐sized potatoes were randomly selected and arranged in three layers inside a cylinder (Figure 3). Given the eight loading time treatments for triaxial and uniaxial tests, the experiments were performed at three replications via triaxial test and 11 replications via uniaxial test and totally 513 potatoes samples were examined. The constant force of 72.5 N was applied to the samples for both triaxial and uniaxial tests. This is equal to a maximum force exerted on a potato gland at bottom layer of a conventional storage with storage height of 1.5 m as bulk. For triaxial tests, we examined the amount of axial deformation of the three central tubers in the three layers (Figure 3). To this end, a rectangular plate with a 40 mm wide and 400 mm long was mounted on top and bottom of the central tuber at middle layer with no contact with the adjacent tubers. A LVDT is mounted on the second plate to measure the deformation rate of the central tuber at the bottom layer. The deformation of the central tuber in the middle layer was measured by a LVDT, which was installed on the first plate from the top surface. The force was applied directly to the middle sample in the top layer through the pressure plate as there was a displacement transducer mounted on pressure plate used to record central tuber deformation at top layer. Figure 4 shows the position of the displacement sensors on the plates in the triaxial test.

**Figure 5:**
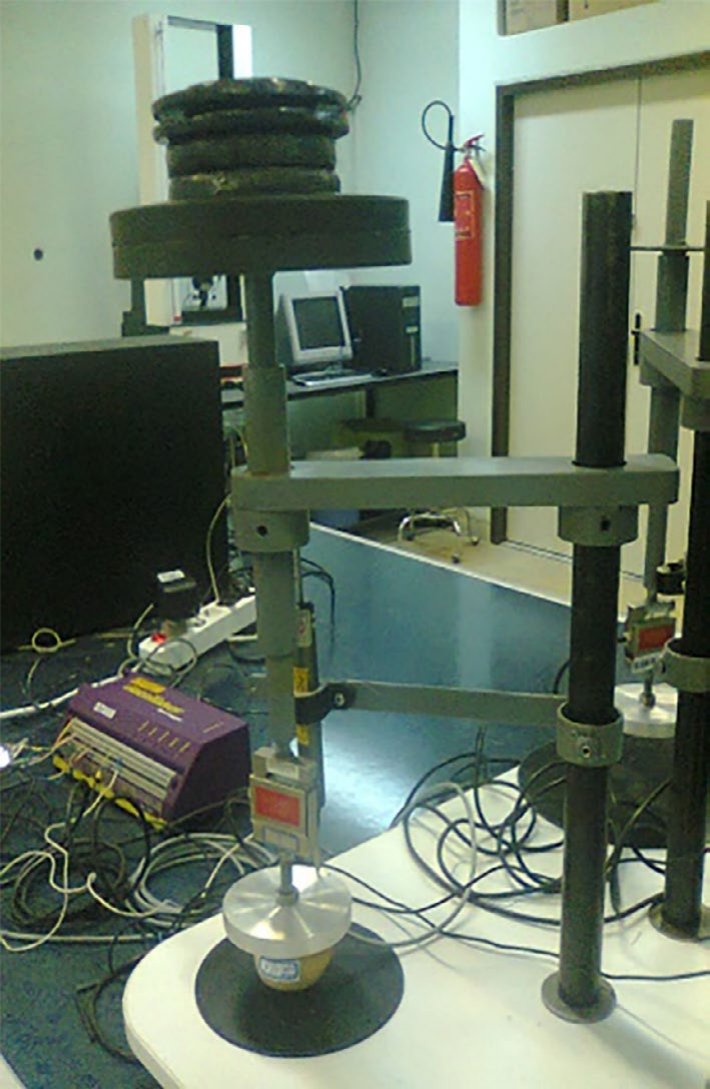
How to Uniaxial creep testing

In the uniaxial test, force is applied to a test specimen and through the pressure plate (Figure 5). A specimen was placed under the pressure plate, and the value of sample deformation relative to time was measured at constant stress using displacement transducer.

The data were recorded simultaneously with the experiment in the data logger, and the recorded data were transferred from data logger to a Laptop. First, the mean data were extracted from the raw data and transferred to SPSS 22 software for the analysis. The data were extracted at times of 5, 30, 60, 120, 240, 360, 420, and 480 min from the entire data. Notably, in conventional storage, the maximum storage time normally is 4–5 months in Iran. However, the main objective of this study is to compare then it was found that 6 hr was sufficient to distinguish their difference. Potato strain was computed by dividing the length of tuber at any time by the initial length of the tuber. Eventually the time‐strain graph was obtained as the output of the device.

## 3 RESULTS AND DISCUSSION

### 3.1 Deformation in different layers of triaxial test

One of the important parameters in the creep test is to know how the specimen should be loaded during the test process. The loading mode must not hit the sample, and stress should back be constant over time.

**Figure 6:**
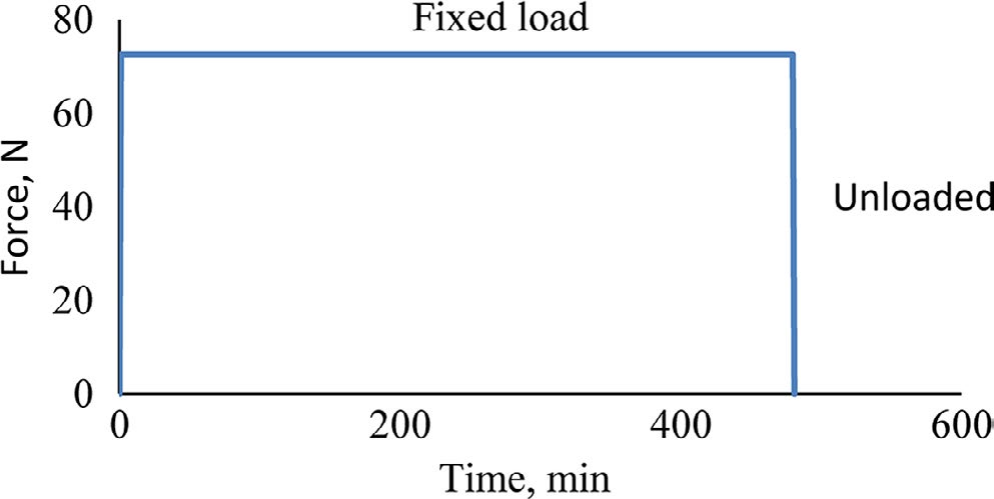
Loading manner of potato samples

Creep phenomena are the deformation of a sample, which is under a constant stress over relatively long time (Alvarez et al., 2000). To provide the process of a test similar to the actual environment, the force applied to the specimen at storage conditions with constant value at different times so that the results can be used in storage and cold storage (Figure 6).

Table 2 shows the results of the deformation of the specimens in the three layers of potato under constant load in triaxial test. Loading was performed on the top layer, and the effect of constant loading on the three layers was investigated. Statistical analysis in Table 2 showed that the deformation value of three different layers was significantly different at the probability level of 1%.

**Table 2:** Results of the analysis of variance of potato deformation with respect to three layers of arrangement

Source of changes	Sum of squares	*df*	Mean of squares	*F* ratio
Treatments	169.221	2	84.611	153.579**
Error	125.611	228	0.551	–
Total	294.832	230	–	–

** Significant at probability level (1%).

**Figure 7:**
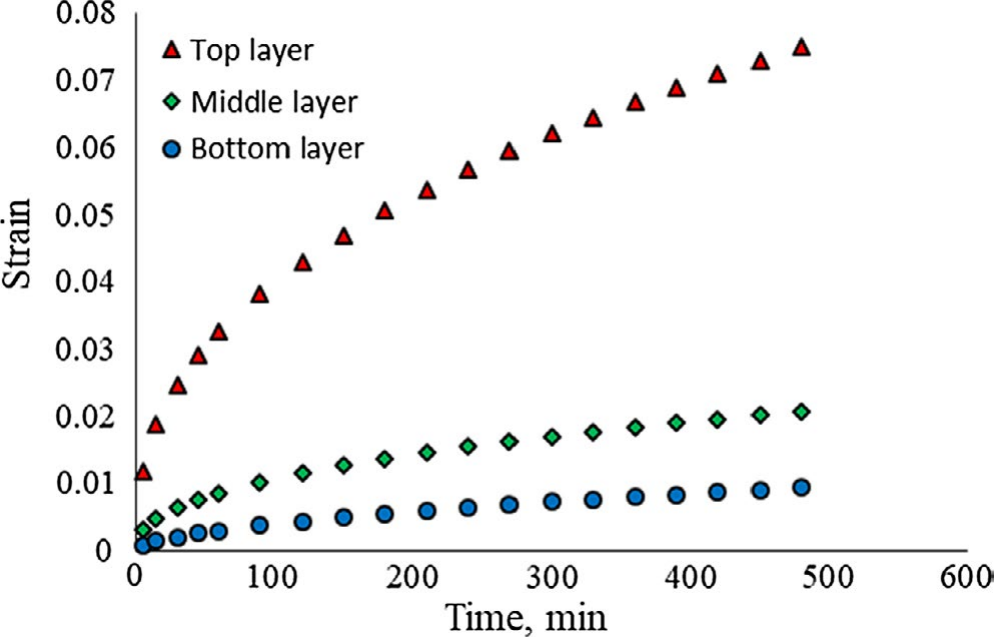
Comparison of the mean deformation of the samples in the three loaded layers over time

It was found that the applied load to the top layer of potatoes influenced the samples of the middle and bottom layer and caused them to be deformed. Unlike metals, viscoelastic materials (e.g., potatoes) absorb some part of applied force at each layer; hence, the applied load to the middle and bottom layers was reduced and accordingly their deformation was less than the upper layer. Figure 7 shows the strain rate of the central sample in the different layers under constant load over time. The loading time increased with the strain of the specimens. After 520 min, the strain of top layer was 4.2 times of middle layer and 10 times of the third layer.

In a research, mechanical damage was evaluated in three layers of two types of apples in a wooden box (Acican, Alibas, & Ozelkok, 2007). The results showed that since the external load was not applied to the samples, it only investigated the influence of the weight of the top layers on the bottom layers. The average mechanical damage in the upper layer was less than the lower layers. Then, the load of the top layers weight affects the lower layers and increases the product waste in the bottom layer. Therefore, it can be concluded if external load is applied together with product weight the maximum deformation occurs in the upper layers and decreases in the lower layers. But if only the weight of upper layers of the product was considered in deformation, greater deformation will occur in lower layers.

### 3.2 Potato deformation over time at constant load in triaxial test

Table 3 illustrates the effect of constant load applied to samples deformation. The experiments were recorded at eight different times including 5, 30, 60, 120, 240, 360, 420, and 480 min. There was a significant difference between the loading times on the samples' strain. That is, the deformation of the viscoelastic specimens increased with the loading time. In a study conducted in Frieden, Isfahan, Iran, the effect of constant loading time on the potato storage was investigated. The results showed that the storage time had a significant effect on the rheological properties of all studied parameters at 5% probability level (Ghasemi, Godarzi, & Hemmat, 2014).

**Table 3:** Results of the analysis of variance of potato deformation relative to the experiment time

Source of changes	Sum of squares	*df*	Mean of squares	*F* ratio
Loading time	52.253	7	7.46	6.88**
Error	242.579	224	1.083	–
Total	294.832	230	–	–

** Significant at probability level (1%).

### 3.3 Potato deformation and strain in uniaxial test

The experiment was conducted on potato crop with 11 replications; the deformation of the product was examined over time under a constant load. The experiments in uniaxial test were also recorded at eight different times of 5, 30, 60, 120, 240, 360, 420, and 480 min. The statistical analysis showed that loading time was highly significant on the deformation of potato sample (Table 4).

**Table 4:** Analysis of variance of potato deformation over time using uniaxial test

Source of changes	Sum of squares	*df*	Mean of squares	*F* ratio
Loading time	54.238	7	7.74	26.68**
Error	8.115	25	0.290	–
Total	62.353	31	–	–

** Significant at probability level (1%).

**Figure 8:**
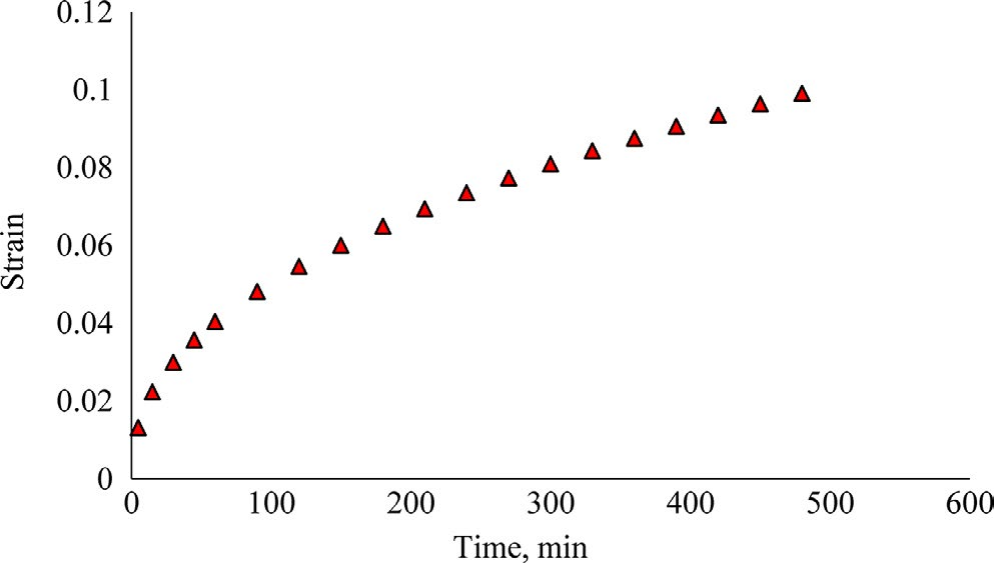
Mean strain of samples relative to time in uniaxial test

Figure 8 shows the sample strain over time. The mean strain at times 5, 30, 60, 120, 240, 360, and 480 were 0.0132, 0.02994, 0.04038, 0.0545, 0.0744, 0.0748, and 0.09904, respectively. When viscoelastic materials are subjected to constant load relative to time, the deformations occur in stages of the instantaneous elastic and the retarded elastic phases. The creep curve can be divided into three main parts: instantaneous elastic, retarded elastic, and Newtonian flow. The total strain is equal to the strains of three instantaneous elastic segments, retarded elastic strain and Newtonian flow (Yong‐Liang, Xiong, Yun‐Bo, & Zhao, 2008). In the fresh potatoes in relation to the stored potatoes because cells have more water, then they have a higher compressibility and less instantaneous strain than the potatoes stored for long time at the same loading. In other words, when the product moisture content reduces, its viscosity value is also decreased. Therefore, more force is required to create a certain displacement relative to fresh product (Khazaei & Mann, 2005). The retarded elastic coefficient was 7 times as large as the instantaneous elasticity coefficient, which indicates that potatoes in the retarded elastic segment have higher elasticity. Therefore, the potato strain in the retarded elastic portion was less than the instantaneous elastic segment. The viscosity of the Newtonian current was 66 times greater than the viscosity of the elastic part. This indicates less product fluidity in the retarded elastic phase (Solomona & Jindal, 2007).

Figure 8 shows that the strain increases with the loading time. In the early stages of loading, the deformation has a higher slope, but slope of deformation increment gradually decreased. Similar trend in the results was obtained by Soliman and EL‐Sayed (2017). The reason for decrement of the deformation slope is reaching to Newtonian flow of the samples.

### 3.4 Comparison of potato strain over time in two triaxial and uniaxial tests

**Figure 9:**
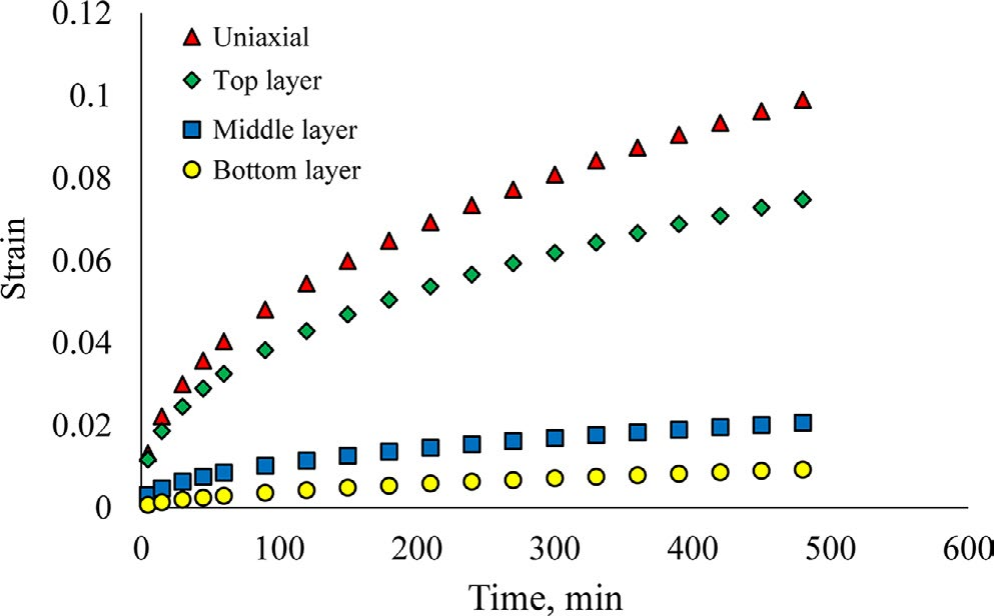
Comparison of strain of samples in two uniaxial and triaxial tests

Figure 9 shows the strain of the specimens that were examined by uniaxial and triaxial tests. At different times with similar applied load, the deformity in the uniaxial test was more than triaxial one. Also in the triaxial test, the deformation decreased from the top to the middle and the bottom layers, respectively. As a triaxial test is close to the actual conditions of the storage, it applies pressure to all the sides of the gland, which hinders the gland from straining too much. However, in the uniaxial state, around the tuber is free and due to axial load much deformation occurs on the sample along the load. Accordingly, it is best that the potato creep test be conducted triaxially to show potato behavior during storage period. In general, potato deformation using uniaxial test was 33% more that potato strain at top layer compared to triaxial test.

## 4 CONCLUSION

The general results of triaxial and uniaxial creep test of potato tubers are as follows:

Triaxial creep test showed that applying loading force on top layer maximum deformation occurred at top layer and deformation rate decreased in lower layers. After 480 min, the strain of top layer was 4.2 times of middle layer and 10 times of the third layer. It was concluded that each layer absorbed some parts of the applied force and reduced it in sublayers. In uniaxial state, tuber was free and due to axial load much deformation occurred along the load. At the same loading, the mean deformation of potato in the uniaxial test was more than triaxial test. It was found that the potato deformation using uniaxial test was 33% more that potato strain at top layer compared to triaxial test. Triaxial creep test evaluates the overall load conditions better than the uniaxial test. Accordingly, it is better the potato creep test be conducted triaxially to show potato behavior during storage period.

## CONFLICT OF INTEREST

The authors have declared no conflict of interest.

## ETHICAL APPROVAL

This study does not involve any human or animal testing.

## INFORMED CONSENT

Written informed consent was obtained from all study participants.
